# Supplementary material for: Rootstocks with Different Tolerance Grade to Citrus Tristeza Virus Induce Dissimilar Volatile Profile in Citrus sinensis and Avoidance Response in the Vector Aphis gossypii Glover
Source: Plants (Basel). 2022 Dec 8;11(24):3426. doi: 10.3390/plants11243426 (PMC9788239; doi:10.3390/plants11243426)
Supplement: Supplementary file 1 [file plants-11-03426-s001.zip › plants-2069925-supplementary.pdf]

plants

MDPI

Supplementary material

Table S1 Tukey multiple pairwise-comparison values of behavioral bioassays among scion/rootstock combinations studied. CS: *Citrus sinensis*; CV: Volkamer lemon; FO: For-ner-Alcaide no. 5; CC: Carrizo citrange; CA: *Citrus aurantium*.

Samples	diff	lwr	upr	*p value*	*p adj*	*p signif*
CC+CS *vs* CA+CS	-4.75	-8.795033	-0.70496732	0.0033	0.01	**
CV+CS *vs* CA+CS	-4.00	-8.045033	0.04503268	0.0121	0.05	*
FO+CS *vs* CA+CS	-4.00	-8.045033	0.04503268	0.0121	0.05	*
CV+CS *vs* CC+CS	0.75	-3.295033	4.79503268	0.626	0.96	ns
FO+CS *vs* CC+CS	0.75	-3.295033	4.79503268	0.626	0.96	ns
FO+CS *vs* CV+CS	0.00	-4.045033	4.04503268	1	1.00	ns

diff: difference between means of the two groups

lwr, upr: the lower and the upper end point of the confidence interval at 95% (default)

p adj (Bonferroni): p-value after adjustment for the multiple comparisons.

** p < 0.05: ** p< 0.01*

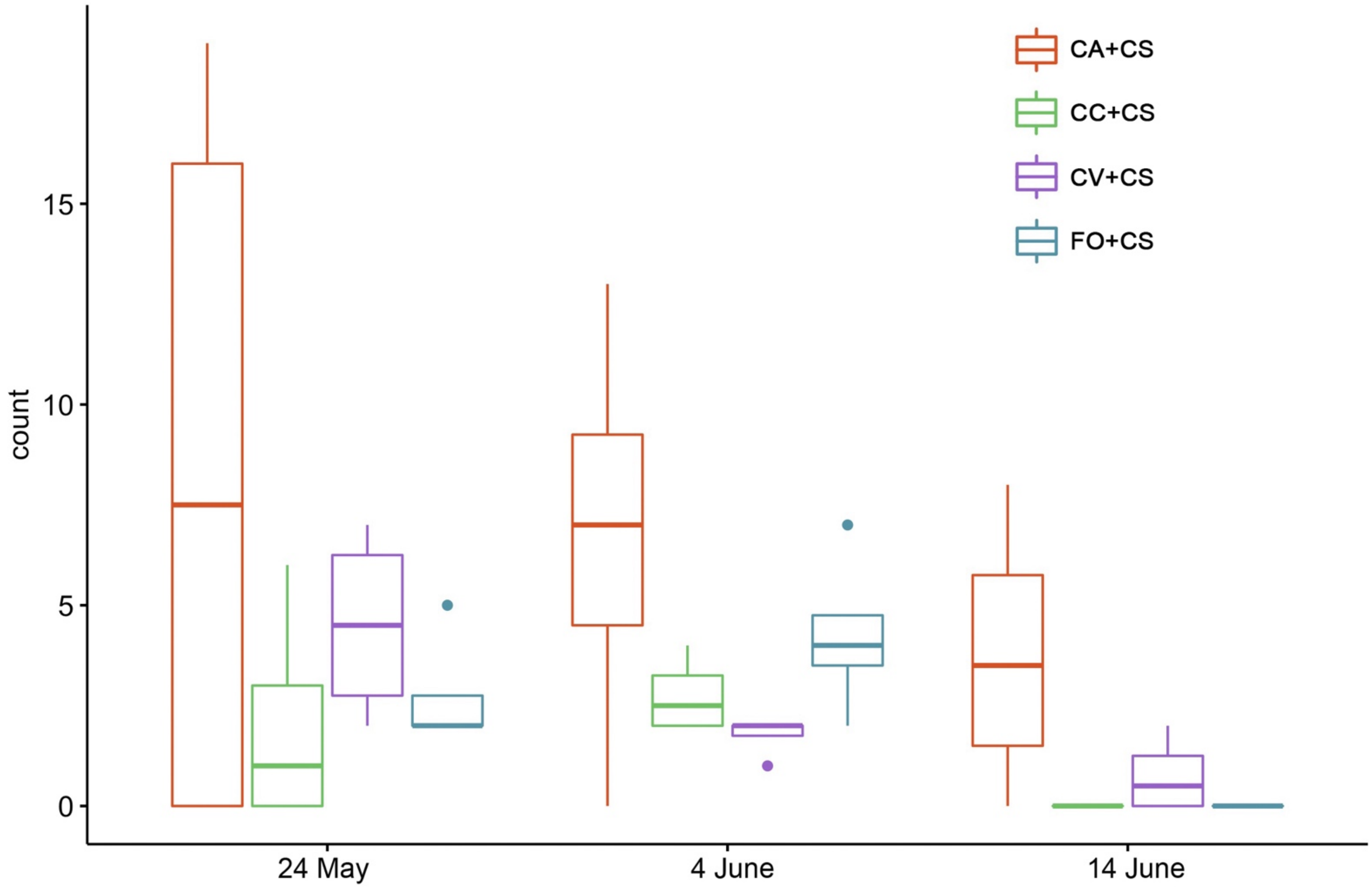

Figure S1 Behavioral bioassays developed on the *Citrus sinensis* (CS) plants grafted on the four different rootstocks studied. The number of infested leaves in *Citrus sinensis* plants grafted on different rootstocks for each timepoint was reported. CV: Volkamer lemon; FO: Forner-Alcaide no. 5; CC: Carrizo citrange; CA: *Citrus aurantium*.

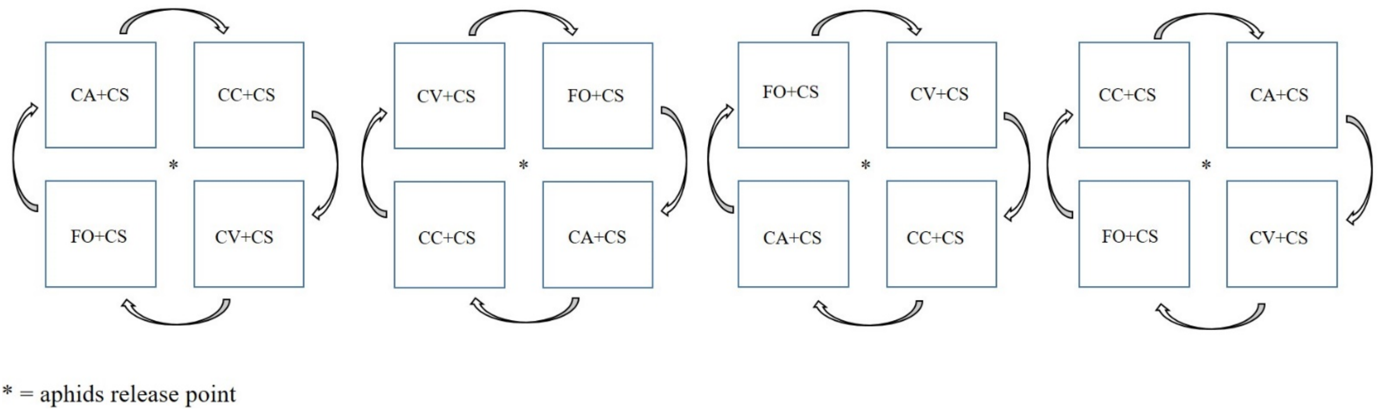

Figure S2 Schematic draw of the cage, size 2.00 x 1.05 x 1.60 m, used for the behavioral bioassays (upper view) on scion/rootstock combinations investigated. CS: *Citrus sinensis*; CV: Volkamer lemon; FO: Forner-Alcaide no. 5; CC: Carrizo citrange; CA: *Citrus aurantium*.
